# *PAI-1* -675 4G/5G Polymorphism in Association with Diabetes and Diabetic Complications Susceptibility: a Meta-Analysis Study

**DOI:** 10.1371/journal.pone.0079150

**Published:** 2013-11-05

**Authors:** Kuanfeng Xu, Xiaoyun Liu, Fan Yang, Dai Cui, Yun Shi, Chong Shen, Wei Tang, Tao Yang

**Affiliations:** 1 Department of Endocrinology, the First Affiliated Hospital with Nanjing Medical University, Nanjing, Jiangsu, China; 2 Department of Epidemiology and Biostatistics, School of Public Health, Nanjing Medical University, Nanjing, Jiangsu, China; 3 Department of Endocrinology, Jiangyin People’s Hospital, Wuxi, Jiangsu, China; Central China Normal University, China

## Abstract

A meta-analysis was performed to assess the association between the *PAI-1* -675 4G/5G polymorphism and susceptibility to diabetes mellitus (DM), diabetic nephropathy (DN), diabetic retinopathy (DR) and diabetic coronary artery disease (CAD). A literature-based search was conducted to identify all relevant studies. The fixed or random effect pooled measure was calculated mainly at the allele level to determine heterogeneity bias among studies. Further stratified analyses and sensitivity analyses were also performed. Publication bias was examined by the modified Begg’s and Egger’s test. Twenty published articles with twenty-seven outcomes were included in the meta-analysis: 6 studies with a total of 1,333 cases and 3,011 controls were analyzed for the *PAI-1* -675 4G/5G polymorphism with diabetes risk, 7 studies with 1,060 cases and 1,139 controls for DN risk, 10 studies with 1,327 cases and 1,557 controls for DR and 4 studies with 610 cases and 1,042 controls for diabetic CAD risk respectively. Using allelic comparison (4G vs. 5G), the *PAI-1* -675 4G/5G polymorphism was observed to have no significant association with diabetes (REM OR 1.07, 95% CI 0.96, 1.20), DN (REM OR 1.10, 95% CI 0.98, 1.25), DR (REM OR 1.09, 95% CI 0.97, 1.22) or diabetic CAD risk (REM OR 1.07, 95% CI 0.81, 1.42), and similar results were obtained in the dominant, recessive and co-dominant models. Our meta-analyses suggest that the PAI-1 -675 4G/5G polymorphism might not be a risk factor for DM, DN, DR or diabetic CAD risk in the populations investigated. This conclusion warrants confirmation by further studies.

## Introduction

The plasminogen activator inhibitor-1 (PAI-1) belongs to the serine protease inhibitor superfamily, and plays a key role in the regulation of extracellular matrix degradation [1]. Studies have indicated that the increase of PAI-1 level was related to the incidence of diabetes mellitus (DM) and its complications, such as an increased risk of diabetic nephropathy (DN), diabetic retinopathy (DR) and diabetic coronary artery disease (CAD), *etc* [2-5]. 

Numerous single nucleotide polymorphisms (SNPs) have been observed in the *PAI-1* gene. Some increase the t^½^ of *PAI-1* while others inactivate it or slow down its secretion into the plasma. It has been found that the most-studied 4G/5G polymorphism (rs1799889), characterized by a single guanosine nucleotide insertion/deletion variation at -675 bp of the *PAI-1* promoter, increases *PAI-1* concentration or its activity in the plasma of humans without changing its t^½^ [6]. This polymorphism is a cause of high plasma *PAI-1* level in 4G/4G allele carriers suggesting that the *PAI-1* 4G/5G polymorphism is a genetic risk factor for diabetes [7]. Recently many studies reported the association between the 4G/5G polymorphism of *PAI-1* gene and the risk of diabetes and diabetic complications [8-27], mainly focusing on DN, DR and diabetic CAD. Despite strong functional evidence for the relevance of several studies, the results for the association with diabetes and its complications showed significant between-study variations and were inconclusive. 

Considering a single study may lack the power to provide a reliable conclusion, we performed a meta-analysis on these eligible case-control studies, to investigate the precise relationship between the *PAI-1* -675 4G/5G polymorphism and susceptibility to DM, DN, DR and diabetic CAD, which would have a much greater possibility of reaching reasonably strong conclusions. 

## Methods

This meta-analysis is reported in accordance with the Preferred Reporting Items for Systematic Reviews and Meta-analyses (PRISMA) statement [28].  

### Search strategy

The comprehensive literature searches were carried out independently by two investigators (Kuanfeng Xu and Xiaoyun Liu) using the electronic data-bases PubMed, Embase and ScienceDirect without language restrictions, and updated on July 11, 2013. The *PAI-1* -675 4G/5G polymorphism was investigated by combinations of the following search terms: ‘plasminogen activator inhibitor-1 or *PAI-1*’, ‘polymorphism, variant or mutation’ and ‘diabetes or diabetic complications’. We used the PubMed option ‘Related Articles’ for each study to retrieve additional potentially relevant articles, and also hand-searched the included articles to identify any other relevant citations. No restriction was set on the source of control participants (general population, clinic or hospital). 

### Inclusion and exclusion criteria

The inclusion and exclusion criteria were as follows: (1) each case-control study were published as an original study designed to evaluate the association; (2) numbers in case and control groups were reported for each allele or genotype; (3) case-control studies had sufficient published data to estimate an odds ratio (OR) with 95% confidence intervals (CI) or provide raw data that allowed us to calculate them; (4) only the most recent or complete study was used if the data were duplicated and had been published in several publications; (5) studies were excluded if the genotype distribution of the controls deviated from Hardy-Weinberg equilibrium (HWE); (6) the following were excluded: animal studies, review articles, abstracts, editorials, reports with incomplete data, studies based on pedigree data or prospective studies, *etc*. 

### Data extraction

Data were independently extracted by two investigators who discussed disagreements and reached a consensus on all of the items. Information extracted from each study was considered as follows: name of first author, year of publication, ethnic origin of the studied population, available number of participants in case and control groups, genotype and allele frequency by case/control status, and OR (95% CI). Not all papers reported the necessary statistics directly, so in some instances we transformed and estimated an OR from the reported data [29]. We did not define any minimum number of patients for a study to be included in our meta-analysis. In addition, all participants of the included studies provided informed consent and the studies were approved by the ethics committees of the participating institutions.

### Statistical analysis

The association of the *PAI-1* -675 4G/5G polymorphism with diabetes and diabetic complications was estimated by calculating the pooled OR and 95% CI in the allelic and genotypic (dominant, recessive and co-dominant model) comparisions respectively. The significance of the OR was determined by the Z test (*P* <0.05 was considered statistically significant). HWE of the genotype distribution of controls was tested by a goodness-of-fit χ^2^ analysis. The distribution was considered to be deviated from HWE at *P* < 0.05.

We employed the DerSimonian and Laird random effect model (REM) to bring the individual effect size estimates together, and quantified between-study heterogeneity by inconsistency index (*I* ^*2*^) statistic. The *I* ^2^ statistic is defined as the percentage of the observed between-study variability that is due to heterogeneity rather than chance, with high values suggesting more possible existence of heterogeneity. Potential heterogeneity between results of individual studies or in subgroups respectively by ethnicity, study design, source of controls, genotyping method, diagnostic criterion, and sample size was explored using χ^2^ test. Sensitivity analysis was conducted to evaluate the key studies with a substantial impact on between-study heterogeneity. Influence analysis was performed to assess the stability of the results, with a single study in the meta-analysis being deleted each time to reflect the influence of the individual data set on the pooled OR. 

Publication bias was assessed by the modified Begg’s and Egger’s test [30]. The significance of the intercept was determined by the *t* test suggested by Egger, with *p* <0.10 considered representative of statistically significant publication bias. All statistical analyses were conducted using RevMan 5.0 (The Nordic Cochrane Centre, The Cochrane Collaboration) & STATA 11.0 (Stata, College Station, TX, USA). All tests were two-sided.

## Results

### Characteristics of study

The trial flow is summarised in Figure S1. A total of 20 published articles with 27 outcomes met the inclusion and exclusion criteria [8-27]. All were case-control studies and most were population-based. The allele and genotype distributions in the studies included are summarised in Table 1. Here 6 studies with a total of 1,333 cases and 3,011 controls were analyzed for the *PAI-1* -675 4G/5G polymorphism with diabetes risk [11,12,19,22,24,27], 7 studies with 1,060 cases and 1,139 controls for this polymorphism with DN [11-14,19,23,26,], 10 studies with 1,327 cases and 1,557 controls for DR [9-11,14,15,18-21,25] and 4 studies with 610 cases and 1,042 controls for diabetic CAD risk [8,16,17,20] respectively. 

**Table 1:** Characteristics of the PAI-1 -675 4G/5G polymorphism allelic and genotype distribution for diabetes and diabetic complications risk in studies included in the meta-analysis.

Study details			DM & complications	Total/Genotypes(4G4G/4G5G/5G5G)		5G allele frequency (%)		OR(95% CI) ^a^
Authors [ref.]	Year	Ethnicity		Cases	Controls	Cases	Controls	
Mansfield MW [8]	1995	European	CAD	38(20/15/3)	122(37/67/18)	27.6	42.2	1.91(1.09-3.36)
Nagi DK [9]	1997	Pima Indian	DR	70(14/44/12)	102(18/45/39)	48.6	60.3	1.61(1.04-2.48)
Broch M [10]	1998	European	DR	82(17/46/19)	95(19/48/28)	51.2	54.7	1.15(0.76-1.75)
Kimura H [11]	1998	Asian	DM	208(64/116/28)	177(66/80/31)	41.3	40.1	0.95 (0.71- 1.27)
			DN	98(28/58/12)	110(36/58/16)	41.8	40.9	0.96(0.65-1.42)
			DR	110(32/62/16)	98(32/54/12)	42.7	39.8	0.89(0.60-1.31)
De Cosmo S ^b^ [12]	1999	European	DM	311(82/156/73)	200(54/96/50)	48.6	49.0	1.02(0.79-1.31)
			DN	175(52/81/42)	136(30/75/31)	47.1	50.4	1.14 (0.83- 1.56)
Tarnow L ^b^ [13]	2000	European	DN	197(54/104/39)	191(63/80/48)	46.2	46.1	1.00(0.75-1.32)
Wong TY [14]	2000	Asian	DN	95(39/37/19)	46(8/29/9)	39.5	51.1	1.60(0.97-2.64)
			DR	84(31/38/15)	57(16/28/13)	40.5	47.4	1.32(0.82-2.14)
Globocnik-Petrovic M [15]	2003	European	DR	124(39/58/27)	80(25/40/15)	45.2	43.8	0.94(0.63-1.14)
Lopes C [16]	2003	European	CAD	229(71/114/44)	406(106/203/97)	44.1	48.9	1.21(0.96-1.53)
Petrovic D [17]	2003	European	CAD	154(45/74/35)	194(68/89/37)	46.8	42.0	0.83(0.61-1.12)
Santos KG [18]	2003	European	DR	99(24/41/34)	111(22/59/30)	55.1	53.6	0.94(0.64-1.39)
Liu SQ [19]	2004	Asian	DM	147(42/75/30)	26(4/16/6)	45.9	53.8	1.37 (0.76- 2.48)
			DN	77(30/28/19)	70(12/47/11)	42.9	49.3	1.30(0.82-2.05)
			DR	56(15/26/15)	91(27/49/15)	50.0	43.4	0.77(0.48-1.23)
Zietz B [20]	2004	European	DR	131(48/55/28)	358(112/173/73)	42.4	44.6	1.09(0.82-1.45)
			CAD	189(59/87/43)	320(108/151/61)	45.8	42.7	0.88(0.68-1.14)
Murata M [21]	2004	Asian	DR	188(78/86/24)	92(43/35/14)	35.6	34.2	0.94(0.65-1.36)
Meigs JB [22]	2006	European	DM	216(65/103/48)	1953(529/995/429)	46.1	47.4	1.06(0.87-1.29)
Martin RJ ^b^ [23]	2007	European	DN	222(70/114/38)	361(111/179/71)	42.8	44.5	1.07(0.84-1.36)
Saely CH [24]	2008	European	DM	148(44/78/26)	524(148/253/123)	43.9	47.6	1.16(0.90-1.50)
Ezzidi I [25]	2009	European	DR	383(77/167/139)	473(54/242/177)	58.1	63.0	1.23(1.01-1.49)
Prasad P [26]	2010	Asian	DN	196(57/90/49)	225(52/117/56)	48.0	50.9	1.12(0.86-1.74)
Al-Hamodi Z [27]	2012	Asian	DM	303(76/151/76)	131(30/63/38)	50.0	53.1	1.13(0.85-1.51)

Note: DM, diabetes mellitus; OR(95% CI), odds ratio (95% confidence intervals); CAD, coronary artery disease; DR, diabetic retinopathy; DN, diabetic nephropathy; a, data were analyzed in the risk allele (4G vs. 5G); b, the data were on the association with type 1 diabetes, others were with type 2 diabetes. The genotype distributions in the controls of all the included studies were in agreement with Hardy-Weinberg equilibrium.

### Quantitative syntheses

**Figure 1:**
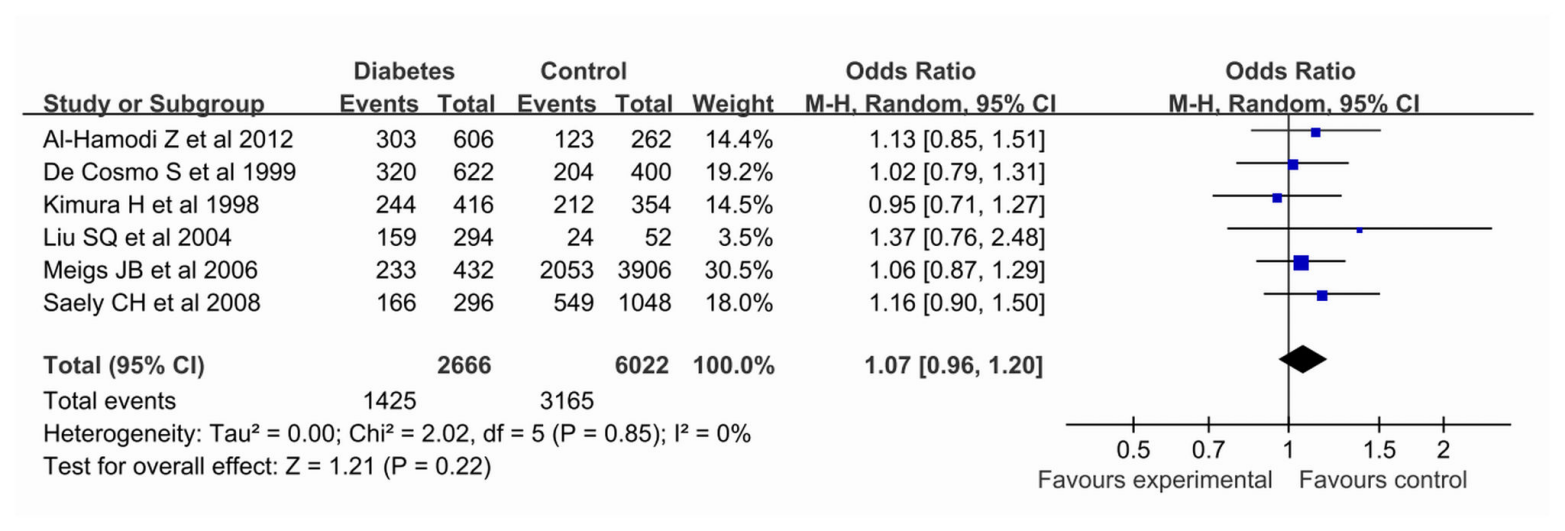
Pooled ORs for the association between the *PAI-1* -675 4G/5G polymorphism (4G vs. 5G) and susceptibility to diabetes. The area of the squares reflects the study-specific weight. The diamond shows the summary fixed-effects OR estimate from 6 studies.

Our meta-analysis revealed no association between the *PAI-1* -675 4G/5G polymorphism and diabetes risk, either by allelic comparision (REM OR 1.07, 95% CI 0.96, 1.20), dominant (REM OR 1.16, 95% CI 0.96, 1.40), recessive (REM OR 1.04, 95% CI 0.88, 1.24) or co-dominant (REM OR 1.18, 95% CI 0.94, 1.47) models. When the study from De Cosmo S et al on type 1 diabetes (T1D) was excluded [12], the results on type 2 diabetes (T2DM) risk were consistent with above in all genetic models (allelic comparision REM OR 1.09, 95% CI 0.96, 1.23; dominant REM OR 1.18, 95% CI 0.95, 1.46; recessive REM OR 1.06, 95% CI 0.87, 1.28; co-dominant REM OR 1.21, 95% CI 0.94, 1.56). Moreover, after stratified by ethnicity, no association was observed using an allelic comparision (Asian descent REM OR 1.07, 95% CI 0.88, 1.30; European descent REM OR 1.06, 95% CI 0.90, 1.18), the similar results were obtained in other genetic models (data not shown). Results of pooled analyses are summarised in detail in Table 2 & Figure 1.

**Table 2:** Pooled measures for the association between the PAI-1 -675 4G/5G polymorphism and susceptibility to diabetes.

Comparisions	Data	*n*			Heterogeneity		OR (95% CI)	Model	*p*
		Studies	Cases	Controls	*I* ^2^ (%)	*P*			
Allelic comparisions	Overall	6	1333	3011	0	0.845	1.07 (0.96-1.20)	REM	0.234
	Asian	3	658	334	0	0.484	1.07(0.88-1.30)	REM	0.502
	European	3	665	2677	0	0.696	1.06(0.90-1.18)	REM	0.324
Dominant	Overall	6	1333	3011	0	0.835	1.16(0.96-1.40)	REM	0.123
Recessive	Overall	6	1333	3011	0	0.447	1.04(0.88-1.24)	REM	0.655
Co-dominant	Overall	6	654	1508	0	0.896	1.18(0.94-1.47)	REM	0.155

Note: Allelic comparisions, 4G vs. 5G; REM, Random effect model.

**Figure 2:**
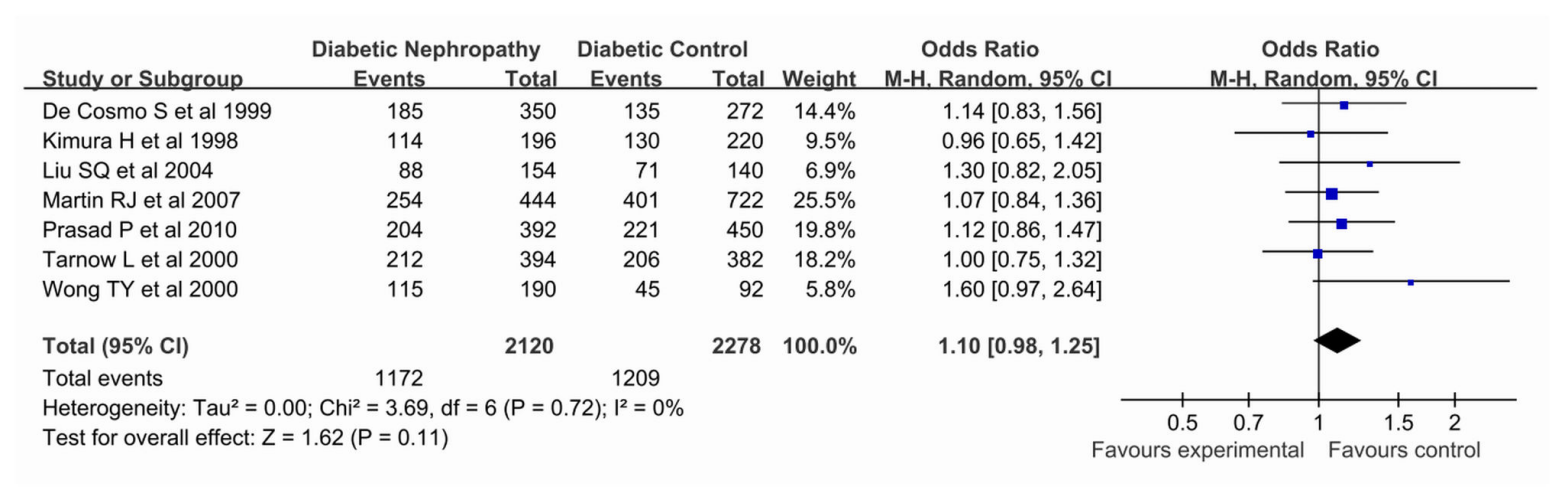
Pooled ORs for the association between the *PAI-1* -675 4G/5G polymorphism (4G vs. 5G) and susceptibility to diabetic nephropathy. The area of the squares reflects the study-specific weight. The diamond shows the summary fixed-effects OR estimate from 7 studies.

Our meta-analysis also showed this polymorphism had no significant association with DN risk using all genetic models (allelic comparision REM OR 1.10, 95% CI 0.98, 1.25; dominant REM OR 1.06, 95% CI 0.86, 1.31; recessive REM OR 1.32, 95% CI 0.93, 1.88; co-dominant REM OR 1.23, 95% CI 0.96, 1.57). Further, no significant association was revealed using an allelic comparision on both T1D (Asian descent, REM OR 1.16, 95% CI 0.97, 1.40) and T2D risk (European descent, REM OR 1.06, 95% CI 0.91, 1.25), and the results were consistent in the dominant, recessive and co-dominant models stratified by ethnicity. Results of pooled analyses are summarised in detail in Table 3 & Figure 2.

**Table 3:** Pooled measures for the association between the PAI-1 -675 4G/5G polymorphism and susceptibility to diabetic nephropathy.

Comparisions	Data	*n*			Heterogeneity		OR (95% CI)	Model	*p*
		Studies	Cases	Controls	*I* ^2^ (%)	*P*			
Allelic comparisions	Overall	7	1060	1139	0	0.763	1.10(0.98-1.25)	REM	0.103
	T1D	4	466	451	0	0.425	1.16(0.97-1.40)	REM	0.108
	T2D	3	594	688	0	0.831	1.06(0.91-1.25)	REM	0.441
Dominant	Overall	7	1060	1139	0	0.682	1.06(0.86-1.31)	REM	0.566
	T1D	4	466	451	0	0.598	0.94(0.68-1.30)	REM	0.699
	T2D	3	594	688	0	0.577	1.16(0.88-1.53)	REM	0.281
Recessive	Overall	7	1060	1139	67.1	0.006	1.32(0.93-1.88)	REM	0.114
	T1D	4	466	451	71.9	0.014	1.72(0.94-3.16)	REM	0.080
	T2D	3	594	688	45.6	0.159	1.04(0.74-1.46)	REM	0.821
Co-dominant	Overall	7	548	554	0	0.933	1.23(0.96-1.57)	REM	0.107
	T1D	4	253	200	0	0.721	1.34(0.90-1.98)	REM	0.148
	T2D	3	295	354	0	0.898	1.16(0.84-1.60)	REM	0.369

Note: Allelic comparisions, 4G vs. 5G; REM, Random effect model. T1D, type 1 diabetes; T2D, type 2 diabetes. Studies on T1D were all from Asian descent, and those on T2D were all from European descent.

**Figure 3:**
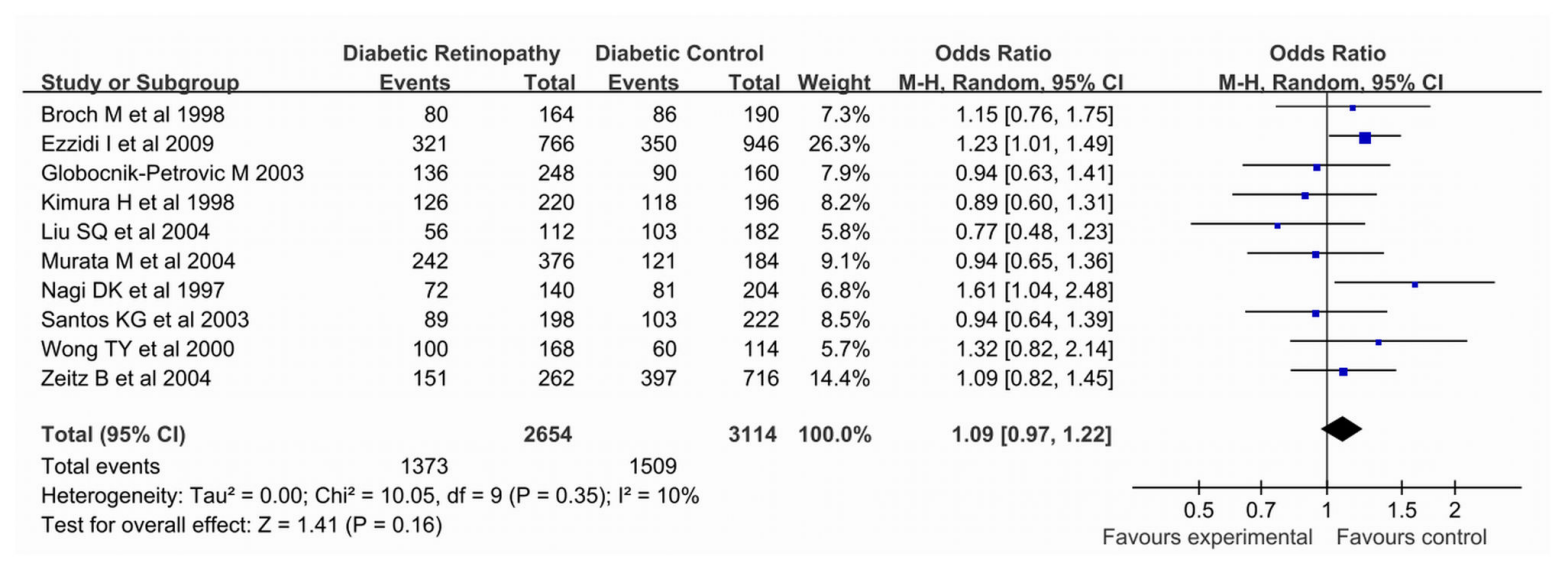
Pooled ORs for the association between the *PAI-1* -675 4G/5G polymorphism (4G vs. 5G) and susceptibility to diabetic retinopathy. The area of the squares reflects the study-specific weight. The diamond shows the summary fixed-effects OR estimate from 10 studies.

On the association with DR risk, the 4G allele was not associated with an increased DR risk using the allelic comparision (REM OR 1.09, 95% CI 0.97, 1.22) or dominant model (REM OR 1.05, 95% CI 0.82, 1.28), the recessive (REM OR 1.18, 95% CI 0.96, 1.46) or co-dominant (REM OR 1.22, 95% CI 0.94, 1.58) model, not using model. Moreover, after stratified by ethnicity, a weak association was revealed by recessive model (REM OR 1.38, 95% CI 1.07, 1.79), but not in an allelic comparision dominant or co-dominant model in populations of European descent, and after Bonferroni correction, the P value in the recessive model were 0.06, which indicated no significant association existed in Europeans. Also our results indicated that no significant association was observed in those of Asian descent in all genetic models. Results of pooled analyses are summarised in detail in Table 4 & Figure 3.

**Table 4:** Pooled measures for the association between the PAI-1 -675 4G/5G polymorphism and susceptibility to diabetic retinopathy.

Comparisions	Data	*n*			Heterogeneity		OR (95% CI)	Model	*p*
		Studies	Cases	Controls	*I* ^2^ (%)	*P*			
Allelic comparisions	Overall	10	1327	1557	10	0.350	1.09(0.97-1.22)	REM	0.160
	Asian	4	438	338	0	0.443	0.95(0.77-1.17)	REM	0.624
	European	5	819	1117	0	0.653	1.12(0.98-1.28)	REM	0.09
Dominant	Overall	10	1327	1557	36.5	0.116	1.05(0.82-1.34)	REM	0.695
	Asian	4	438	338	6.7	0.360	0.94(0.63-1.41)	REM	0.765
	European	5	819	1117	0	0.626	0.99(0.80-1.21)	REM	0.910
Recessive	Overall	10	1327	1557	22.8	0.233	1.18(0.96-1.46)	REM	0.128
	Asian	4	438	338	0	0.560	0.93(0.68-1.26)	REM	0.629
	European	5	819	1117	18.7	0.295	1.38(1.07-1.79)	REM	0.015
Co-dominant	Overall	10	704	784	19.6	0.263	1.22(0.94-1.58)	REM	0.132
	Asian	4	226	172	0	0.413	0.94(0.61-1.46)	REM	0.779
	European	5	452	555	8.4	0.359	1.31(0.98-1.75)	REM	0.068

Note: Allelic comparisions, 4G vs. 5G; REM, Random effect model.

**Figure 4:**
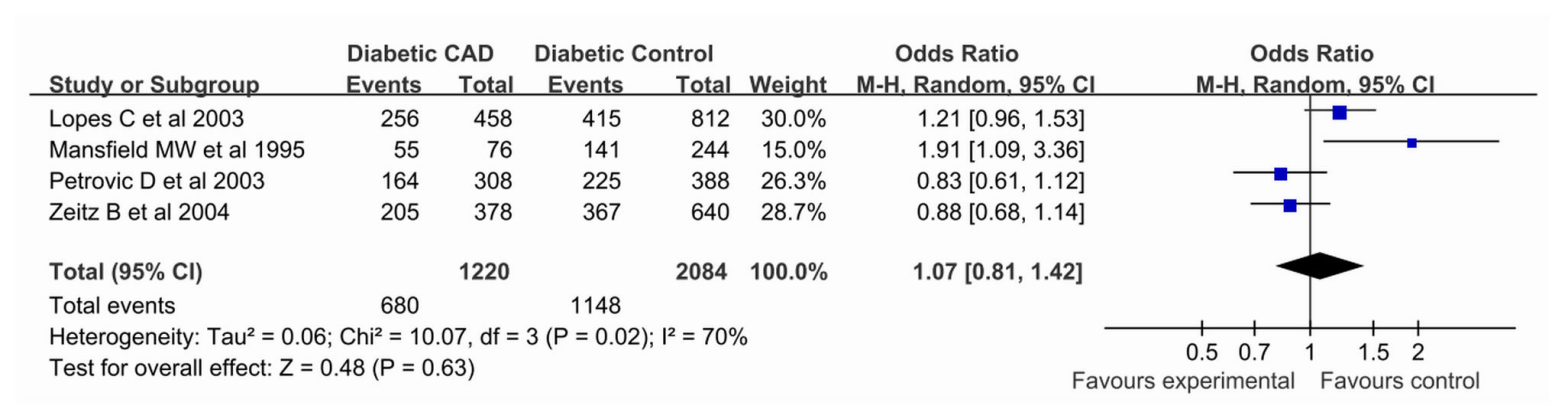
Pooled ORs for the association between the *PAI-1* -675 4G/5G polymorphism (4G vs. 5G) and susceptibility to diabetic coronary artery disease. The area of the squares reflects the study-specific weight. The diamond shows the summary random-effects OR estimate from 4 studies.

In addition, our meta-analysis showed no significant association between the *PAI-1* -675 4G/5G polymorphism and diabetic CAD in all genetic models (allelic comparision REM OR 1.07, 95% CI 0.81, 1.42; dominant REM OR 1.01, 95% CI 0.72, 1.42; recessive REM OR 1.13, 95% CI 0.76, 1.68; co-dominant REM OR 1.08, 95% CI 0.65, 1.80), which were all from European descent. Results of pooled analyses are summarised in detail in Table 5 & Figure 4.

**Table 5:** Pooled measures for the association between the PAI-1 -675 4G/5G polymorphism and susceptibility to diabetic coronary heart diseases.

Comparisions	Data	*n*			Heterogeneity		OR (95% CI)	Model	*p*
		Studies	Cases	Controls	*I* ^2^ (%)	*P*			
Allelic comparisions	Overall	4	610	1042	69.5	0.054	1.07(0.81-1.42)	REM	0.631
Dominant	Overall	4	610	1042	36.2	0.195	1.01(0.72-1.42)	REM	0.954
Recessive	Overall	4	610	1042	67.4	0.027	1.13(0.76-1.68)	REM	0.555
Co-dominant	Overall	4	407	683	63.0	0.044	1.08(0.65-1.80)	REM	0.772

Note: Allelic comparisions, 4G vs. 5G; REM, Random effect model.

### Sensitivity analyses and influence analyses

As shown in Table 2-5, low or high heterogeneity in most of the inherited models was observed among studies in the overall population except for the association with diabetes risk. To identify the studies with the greatest impact on the overall between-study heterogeneity, sensitivity analyses and stratified analyses were conducted. On the association with DN risk, the heterogeneity in the recessive model was not significantly decreased after either sensitivity analysis or stratified analysis by ethnicity. On the association with DR risk, low heterogeneity existed in all genetic models. Sensitivity analysis indicated that the heterogeneity was also effectively decreased in all genetic models when excluded the study from Nagi DK et al [9]. Moreover, when the data were stratified by ethnicity, the heterogeneity in all genetic models was significantly decreased or eliminated in populations of Asian and European descent. On the association with diabetic CAD risk, the study from Mansfield MW et al [8] were mainly responsible for the observed heterogeneity, but sensitivity analysis suggested that the significant heterogeneity still existed in all genetic models when excluded this study. 

To assess the degree to which each individual study affected the overall OR estimates, influence analysis was conducted by repeating the meta-analysis sequentially excluding one study at a time. No single study excessively influenced the analyses (data not shown).

### Publication bias

Funnel plots and Egger’s test were performed to assess the publication bias of the literature. As expected, symmetrical funnel plots were obtained in diabetes and its complications tested in all genetic models. And Egger’s test further confirmed no publication bias for any of the polymorphisms examined, indicating that our results are statistically robust, as shown in Table S1.

## Discussion

Elevated concentrations of *PAI-1* have been observed consistently in blood from patients with T2D or insulin resistance [2,3]. The *PAI-1* -675 4G/5G polymorphism, a single guanosine insertion/deletion, has been identified to contain an additional binding site for a DNA binding protein that may play a pivotal role as a repressor during transcription and exert the greatest impact on plasma PAI-1 concentration [6,31]. The information suggested that this polymorphism might be a genetic risk factor for diabetes. However, our meta-analysis results indicated that the *PAI-1* -675 4G/5G polymorphism had no association with T2D in all genetic models. Further studies also indicated that the elevation of *PAI-1* concentration correlates with complications of diabetes, including DN, DR and diabetic CAD risk [32-34]. Also studies on the *PAI-1* -675 4G/5G polymorphism with the risk of diabetic complications were reported, which showed significant between-study variations and were inconclusive. Unfortunately, in our results of meta-analysis this polymorphism had no association with risk of diabetic complications in overall populations in all genetic models, given that the underlying studies were carried out in different populations, we also performed by random effects model, in which the results indicated no association in either European or Asian descendent.

Heterogeneity is potentially a significant problem when interpreting the results of any meta-analysis of genetic association studies [35]. To determine the amount of heterogeneity that existed among these variants, we did an χ^2^-based Q test. Our meta-analysis showed no significant between-study heterogeneity except for diabetic CAD risk in all genetic models and DN risk in the recessive model. Many of the variables that varied between the various studies might be responsible for this observed heterogeneity, including the source of the controls, sex bias, ethnicity, etc. Initial inspection of the data did not immediately identify any likely candidate variable or study that was significantly impacting on our overall results. Then we conducted sensitivity analyses by repeating the meta-analysis with one study excluded at each time [36]. The results showed that none of the individual study dramatically influenced the heterogeneity or pooled ORs. 

The results of the present meta-analysis should also be interpreted within the context of its limitations. First, other potential environment-gene interactions or gene-gene interactions may well be contributors to the observed disease-effect unconformity except for ethnicity, but we had insufficient data to perform an evaluation of such interactions. Second, our meta-analysis is based on unadjusted estimates because of a lack of original data. Third, though the Egger’s test gave no publication bias, sample size is still a limitation of our meta-analysis [37], especially on the association with diabetic CAD risk. 

In conclusion, our results indicate that the PAI-1 -675 4G/5G polymorphism might not be a risk factor for DM, DN, DR or diabetic CAD risk in the populations investigated. Maybe this association is not robust and could be due to chance, and additional larger studies that allow stratification for other gene-gene and gene-environment should also be conducted in future analyses.

## Supporting Information

Checklist S1 **PRISMA Checklist.**
(DOC)

Figure S1 **Systematic review flow diagram n, number of studies.**
(TIF)

Table S1 **Egger's publication bias test for the PAI-1 -675 4G/5G polymorphisms in DM, DN, DR and diabetic CAD.**
(DOC)
