# Supplementary figures and images for: PAI-1 -675 4G/5G Polymorphism in Association with Diabetes and Diabetic Complications Susceptibility: a Meta-Analysis Study
Source: PLoS One. 2013 Nov 5;8(11):e79150. doi: 10.1371/journal.pone.0079150 (PMC3818463; doi:10.1371/journal.pone.0079150)

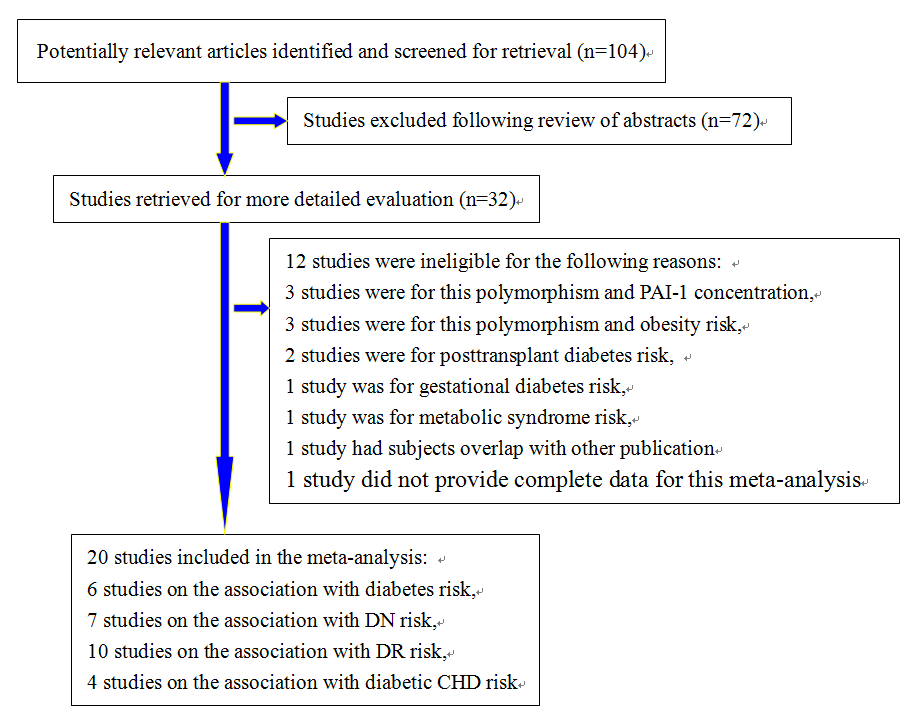

Supplement: Figure S1 — Systematic review flow diagram n, number of studies. (TIF) [file pone.0079150.s002.tif]
