# Supplementary material for: PAI-1 -675 4G/5G Polymorphism in Association with Diabetes and Diabetic Complications Susceptibility: a Meta-Analysis Study
Source: PLoS One. 2013 Nov 5;8(11):e79150. doi: 10.1371/journal.pone.0079150 (PMC3818463; doi:10.1371/journal.pone.0079150)
Supplement: Table S1 — Egger's publication bias test for the PAI-1 -675 4G/5G polymorphisms in DM, DN, DR and diabetic CAD. (DOC) [file pone.0079150.s003.doc]

**Table S1**

| DM &  complications | Comparisions | | | |
| --- | --- | --- | --- | --- |
|  | 4G vs. 5G | Dominant model | Recessive model | Co-dominant model |
| DM | 0.198 | 0.318 | 0.444 | 0.122 |
| DN | 0.137 | 0.297 | 0.076 | 0.153 |
| DR | 0.324 | 0.563 | 0.142 | 0.179 |
| Diabetic CAD | 0.539 | 0.681 | 0.442 | 0.594 |

Note: DM, diabetes mellitus; DN, diabetic nephropathy; CAD, DR, diabetic retinopathy; CAD, coronary artery disease.
